# Comprehensive analysis of non-tumor lung, liver, and kidney transcriptomes in canine metastatic osteosarcoma

**DOI:** 10.1038/s42003-026-09870-x

**Published:** 2026-03-23

**Authors:** Jessica A. Beck, Anjali Garg, Peter L. Choyke, Christina Mazcko, Amy K. LeBlanc

**Affiliations:** 1 Department of Veterinary Biosciences, College of Veterinary Medicine, The Ohio State University, Columbus, OH USA; 2 Comparative Oncology Program, National Cancer Institute, National Institutes of Health, Bethesda, MD USA; 3 Molecular Imaging Branch, National Cancer Institute, NIH, Bethesda, MD USA

**Keywords:** Cancer microenvironment, Bone cancer

## Abstract

The non-tumor tissue adjacent to metastases can appear morphologically unremarkable under a microscope; however, it is exposed to a milieu of secretory factors and proteins derived from tumor cells, stromal cells, and immune cells within the surrounding tumor microenvironment. Studies investigating the peritumoral tissue (PTT) or so-called Normal tissue Adjacent to Tumor tissue (NAT) have identified distinct differences between the genomic and transcriptomic profiles of healthy and tumor-adjacent non-tumor tissues. These alterations are hypothesized to have significant implications in local tumor progression, metastasis, and patient outcome. Most NAT/PTT studies focus on the primary tumor microenvironment (TME) with comparisons between patients with and without cancer. The study described herein expands upon this work by investigating the metastatic TME with comparisons between met-recipient and met-free tissues, both derived from a canine osteosarcoma clinical trial. Our study identifies shared and tissue-specific changes in met-recipient non-tumor lung, liver, and kidney which overlap with transcriptional alterations described in human cancers. These findings improve our understanding of the landscape of the peritumoral TME of metastatic osteosarcoma and further underscore the translational relevance of the canine patient as a model of human disease.

## Abstract (web summary)

Analysis of the transcriptome of lung, liver, and kidney from canine osteosarcoma patients identifies alterations in the non-tumor tissue adjacent to osteosarcoma metastases with overlap to that described in the peritumoral tissue in human cancers.

## Introduction

Osteosarcoma is a rare cancer of bone that primarily affects pediatric and adolescent patients^1^. While local tumor control strategies have a high success rate, there is a critical unmet need for therapies targeting metastatic disease^2^. Cellular and stromal components within the tumor microenvironment (TME) provide therapeutic opportunities for promoting anti-tumor responses; examples include treatments that target pro-tumor mechanisms (e.g., angiogenesis^3^, fibroblasts and fibrosis^4^, and immune checkpoint proteins^5^) or those that enhance anti-tumor activities (e.g., CAR T-cell therapy^6^). A better understanding of the peritumoral TME of metastatic disease will provide important context to mechanisms of metastatic progression in support of efforts to identify novel therapeutic targets for patients with metastatic osteosarcoma.

With the scarcity of human samples in rare but devastating cancers like osteosarcoma, cell-based studies and informative animal models are vital for the advancement of osteosarcoma research^7,8^. Canine patients develop spontaneous osteosarcoma in their natural lifespan with strong biologic similarities to that of human osteosarcoma^9^. The presence of metastases is the primary determinant of outcome in both patient populations. In the dog, approximately 75% of appendicular osteosarcomas will metastasize to the lung, 30% to the kidney, and 15% to the liver^9^. The predilection for metastasis to the lung is also well-documented in human osteosarcoma patients^10^. Without confirmation at autopsy, the incidence of extrapulmonary metastases may be underappreciated in human patients^11^. In canine clinical trials, postmortem examinations are relatively common and allow for the collection of metastases and adjacent non-tumor tissues which facilitates the establishment of biobanks^9^; these multi-institutional efforts support studies investigating the functions of non-tumor tissue in osteosarcoma biology.

Although tissue adjacent to tumors may appear morphologically normal, it demonstrates a variety of subtle changes^12,13^. In human patients, many of the first studies of cancer-adjacent non-tumor tissue were focused on breast cancer^14–16^. Data from The Cancer Genome Atlas Program (TCGA) has also contributed significantly to the advancement of this field through the identification of a pan-cancer signature that highlighted transcriptional changes in the stroma within non-tumor tissue adjacent to cancer^13^. Many studies have aimed to identify features within the non-tumor tissue adjacent to cancer that promote tumor recurrence or predict patient outcomes^15,17–19^. Interestingly, the transcriptional profiles of peritumoral tissues may be more informative for patient outcomes including survival and recurrence as compared to the tumor tissue itself^17,20^.

**Fig. 1:**
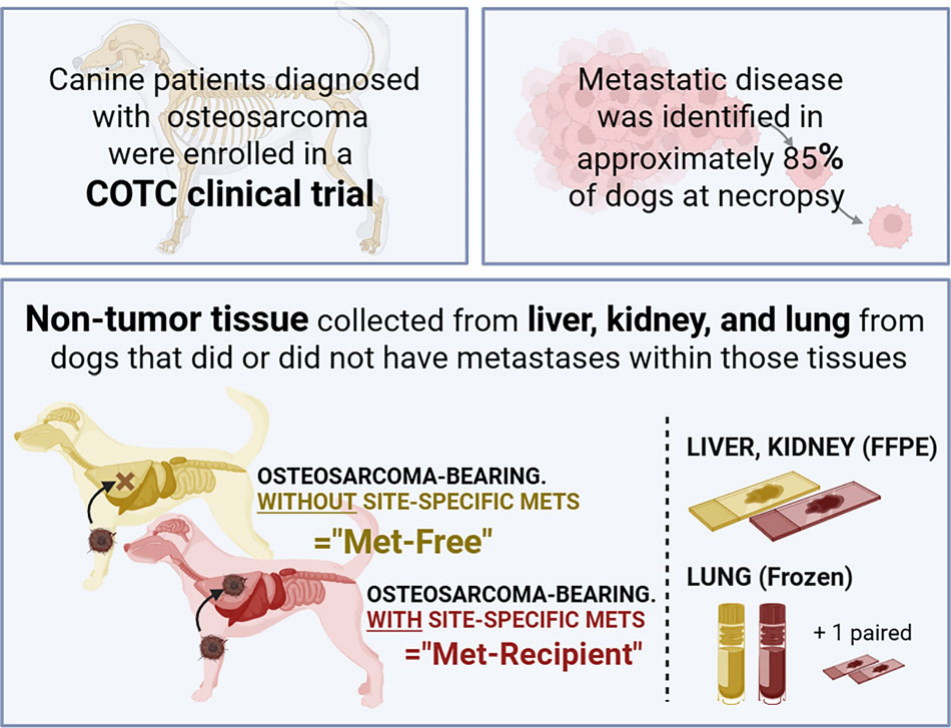
Study outline. Illustration showing the sample types examined in this study. All samples are derived from osteosarcoma-bearing pet dogs. Comparisons are made between non-tumor tissues (lung, liver, kidney) that either lack (met-free) or contain (met-recipient) metastases. Created in BioRender.

In contrast to many of these earlier efforts, our study does not utilize healthy samples from non-tumor bearing individuals but instead compares non-tumor samples taken from canine osteosarcoma patients with and without metastases referred to as met-recipient and met-free tissues, respectively (Fig. 1). Met-free tissues are distinct from healthy controls as they are derived from patients with osteosarcoma. This approach highlights differential gene expression due to the local presence of metastatic tumor rather than the general disease state, which may be impacted by circulating tumor cells, tumor-derived products, and the inflammatory response. In addition, although many studies utilize samples taken from different databases, trials, or populations, all tissues in this study were derived from the same canine clinical trial^21^ thus reducing the potential for confounding variables, including sample preparation or data collection, which can induce batch effects or other biases between patient groups.

In this work, our central hypothesis is that osteosarcoma cells introduce secretory proteins and pro-inflammatory signals into the surrounding non-tumor tissue and that the resulting stromal changes may offer key insight for mechanisms that contribute to tumor progression. We further hypothesized that there is overlap between the peritumoral transcriptome of human and canine patients underscoring the value of the tumor-bearing canine patient in comparative oncology research. Finally, an improved characterization of the transcriptional landscape of non-tumor tissue may elucidate additional therapeutic targets for the treatment of metastatic osteosarcoma, for which no widely effective therapies currently exist.

## Results

### Site-specific and shared alterations in gene expression between met-recipient lung, liver, and kidney in osteosarcoma-bearing pet dogs

**Fig. 2:**
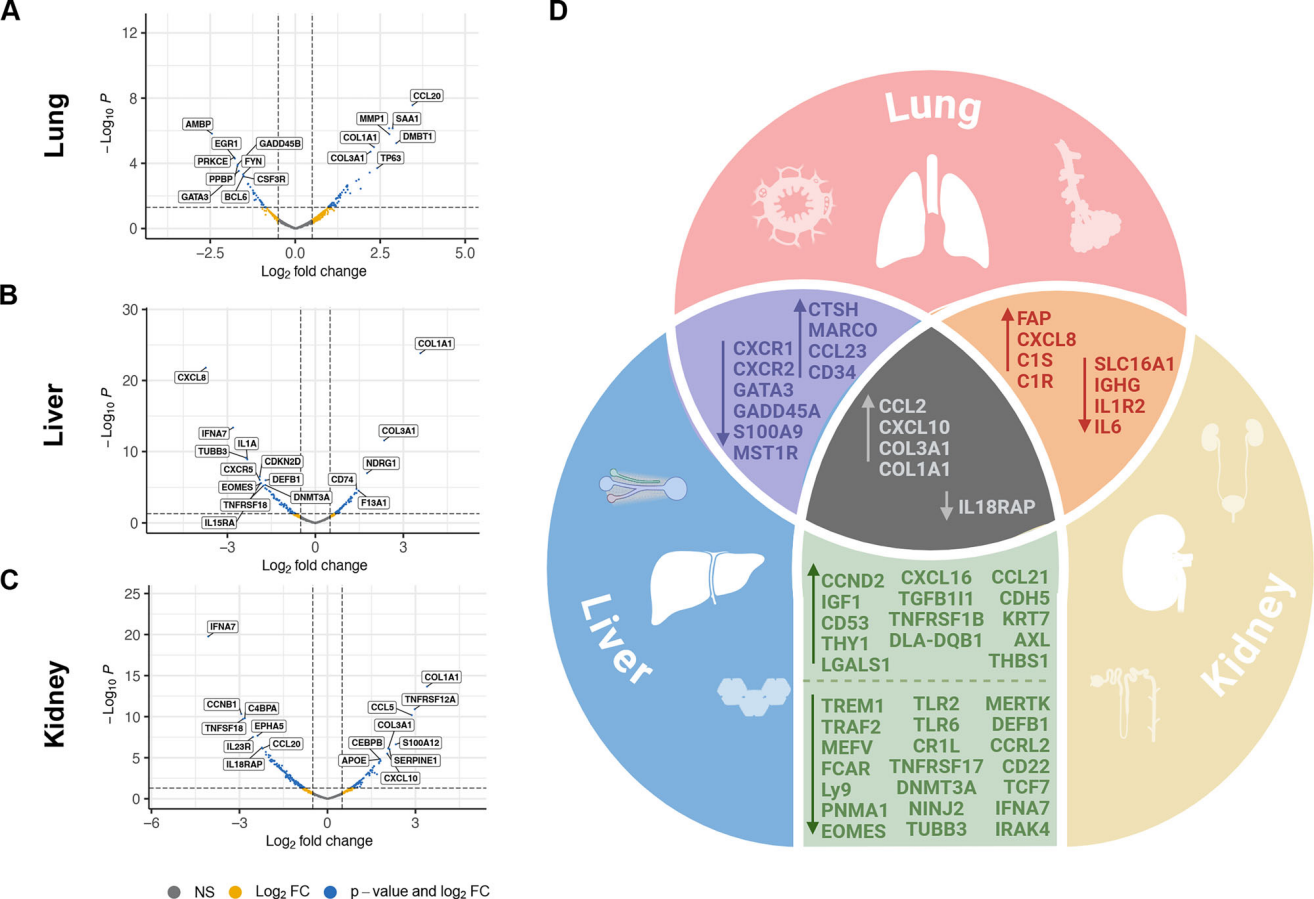
Site-specific and shared alterations in gene expression between met-recipient lung, liver, and kidney in osteosarcoma-bearing pet dogs. Identification of differentially expressed genes in (**A**) Lung, (**B**) Liver, and (**C**) Kidney. **D** Comparison of shared genes either increased or decreased in met-recipient tissues across all three sites. Created in BioRender.

We examined non-tumor tissues derived from osteosarcoma patients with and without metastases in the lung, liver, and kidney which are referred to as met-recipient and met-free tissues, respectively (Fig. 1**;** Supplementary Data 1). Differential gene expression was determined within each tissue site (Fig. 2A–C). Top upregulated genes identified in this analysis included CCL20 in met-recipient lung, and COL1A1 in met-recipient liver and kidney. Downregulated genes included AMBP in met-recipient lung, CXCL8 in met-recipient liver, and IFNA7 in met-recipient kidney. Across all three tissue sites (lung, liver, kidney), there is shared upregulation of genes associated with extracellular matrix and inflammation in met-recipient non-tumor tissue (Fig. 2D; Supplementary Data 2), including chemokines (CCL2/MCP1, CXCL10) and collagen genes (COL3A1, COL1A1). Each met-recipient tissue also demonstrates site-specific changes in gene expression compared to met-free tissues. For example, GFAP is significantly decreased within met-recipient liver (logFC = −1.41; *p* = 0.00013) while SP-C (logFC = 1.52; *p* = 0.0023) is increased within met-recipient lung (Supplementary Data 2). These genes are expressed in tissue specific cell populations, namely hepatic stellate cells^22^ and type II pneumocytes^23^, respectively.

In addition to met-free and met-recipient tissues, several paired samples were analyzed including tissue adjacent and distant to a metastatic lung lesion (Supplementary Fig. 1A) and two renal metastases (Supplementary Fig. 1B). Furthermore, because previous studies have identified differences between geographically-distinct sections of the same organ^13^, we also evaluated the transcriptome of patient-matched renal medulla and cortex which are distinct regions of the kidney with disparate structure and function. This analysis identified enrichment in the apical junction pathway in matched renal cortex suggesting that this may be reflective of differences between renal cortex and medulla rather than changes induced by the absence or presence of osteosarcoma metastases (Supplementary Fig. 2).

### Met-recipient tissues are enriched in fibroblasts, endothelial cells, and monocytes

**Fig. 3:**
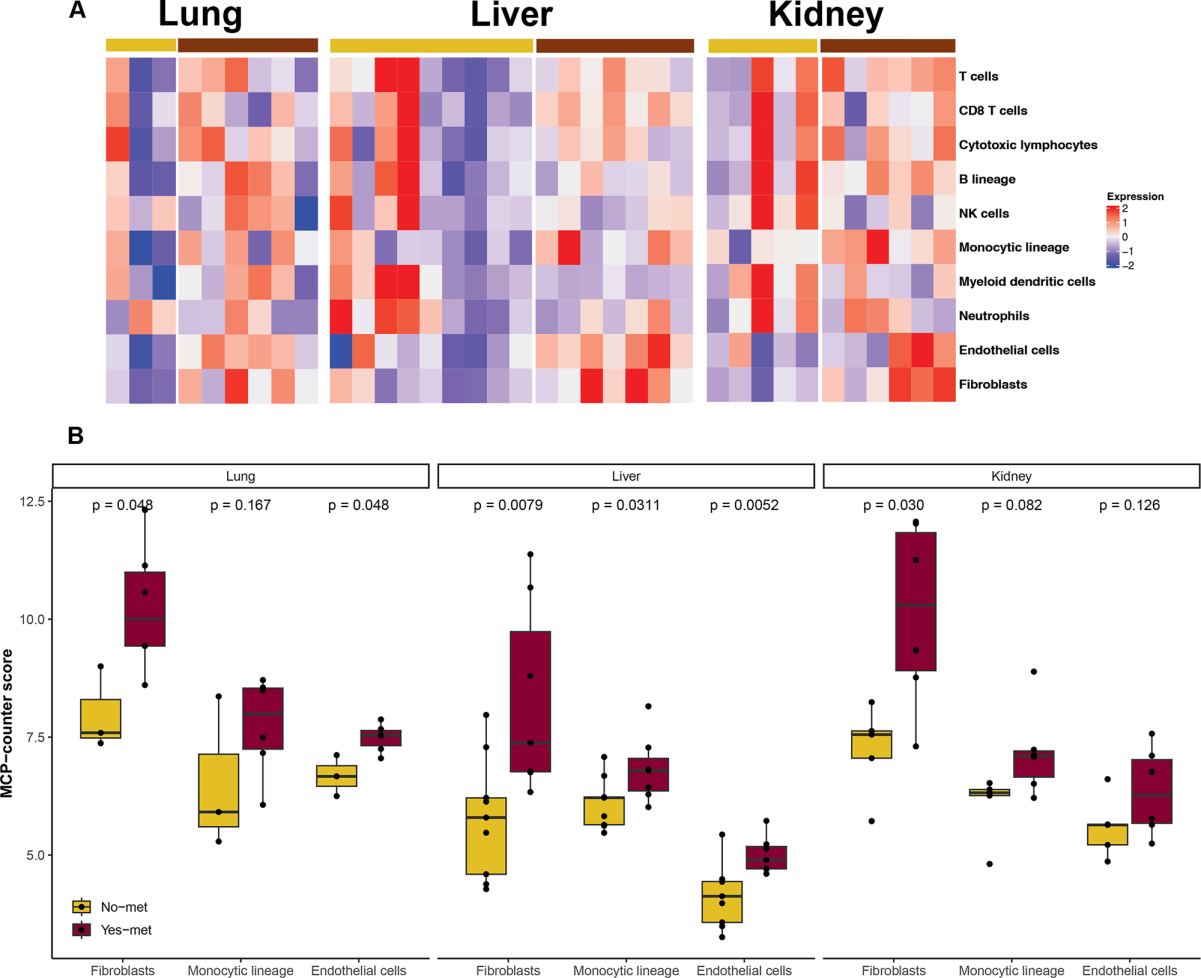
Cell deconvolution analysis in metastatic osteosarcoma. **A** cell deconvolution analysis comparing cell types within met-free (yellow) and met-recipient (red) tissues. **B** Comparison of endothelial, fibroblast, and monocytic cells between met-free (red) and met-recipient (blue) tissues from lung, (**C**) liver, and (**D**) kidney.

Our previous work identified a role for the primary osteosarcoma TME in outcome in canine patients, namely that enrichment in collagen, endothelia and fibroblasts is associated with reduced survival and disease-free interval^24^. Critically, this finding held true in pediatric osteosarcoma patients, underscoring the value of the canine model for identifying prognostic biomarkers with translational relevance. To examine non-tumor tissue of metastatic osteosarcoma patients, we next performed cell deconvolution analysis to compare met-free and met-recipient lung, liver, and kidney (Fig. 3A). Fibroblasts were significantly enriched in met-recipient lung, liver, and kidney (Fig. 3B). Endothelial cells were increased within the lung (*p* = 0.048) and liver (*p* = 0.0052), but not the kidney (*p* = 0.13). Cells of the monocytic lineage were significantly enriched within the met-recipient liver. They were not significantly increased in the kidney (*p* = 0.082) or lung (*p* = 0.17; Supplementary Data 2). Immune cells that were not significantly different between met-free and met-recipient lung, liver, and kidney included T cells, B cells, NK cells, and neutrophils. To further investigate the role of non-tumor cells within the osteosarcoma TME, we examined publicly available spatial transcriptomics from human osteosarcoma metastases^25^ and single-cell RNA sequencing from canine primary osteosarcomas^26^. The cell types positively correlated with each gene varied by sample (Supplementary Fig. 3; Supplementary Data 2). In the human samples, expression of COL1A1 and COL3A1 was associated with fibroblast subtypes, such as alveolar fibroblasts and myofibroblasts; expression was also associated with cells of the monocyte-macrophage lineage. The fibroblast cell type was also associated with COL3A1 (β = 0.5) in the canine primary osteosarcoma samples. COL1A1 was expressed in the canine osteosarcoma TME and was used as a marker of fibroblasts in this dataset. Expression of CXCL10 and CCL2 was limited to canine primary osteosarcoma samples and human lung metastases, respectively. Tumor-associated macrophages (IFN-TAM, β = 0.2; TAM_ACT, β = 0.1) and dendritic cells (cDC1, β = 0.1; mregDC, β = 0.1) were associated with CXCL10 expression in canine samples. In human osteosarcoma metastases within the lung, CCL2 was correlated with multiple cell types including alveolar macrophages, monocytes, myofibroblasts, and plasma cells.

### Compared to the matched non-tumor, met-recipient tissues, metastases are enriched in T cells, NK cells, myeloid dendritic cells and fibroblasts

**Fig. 4:**
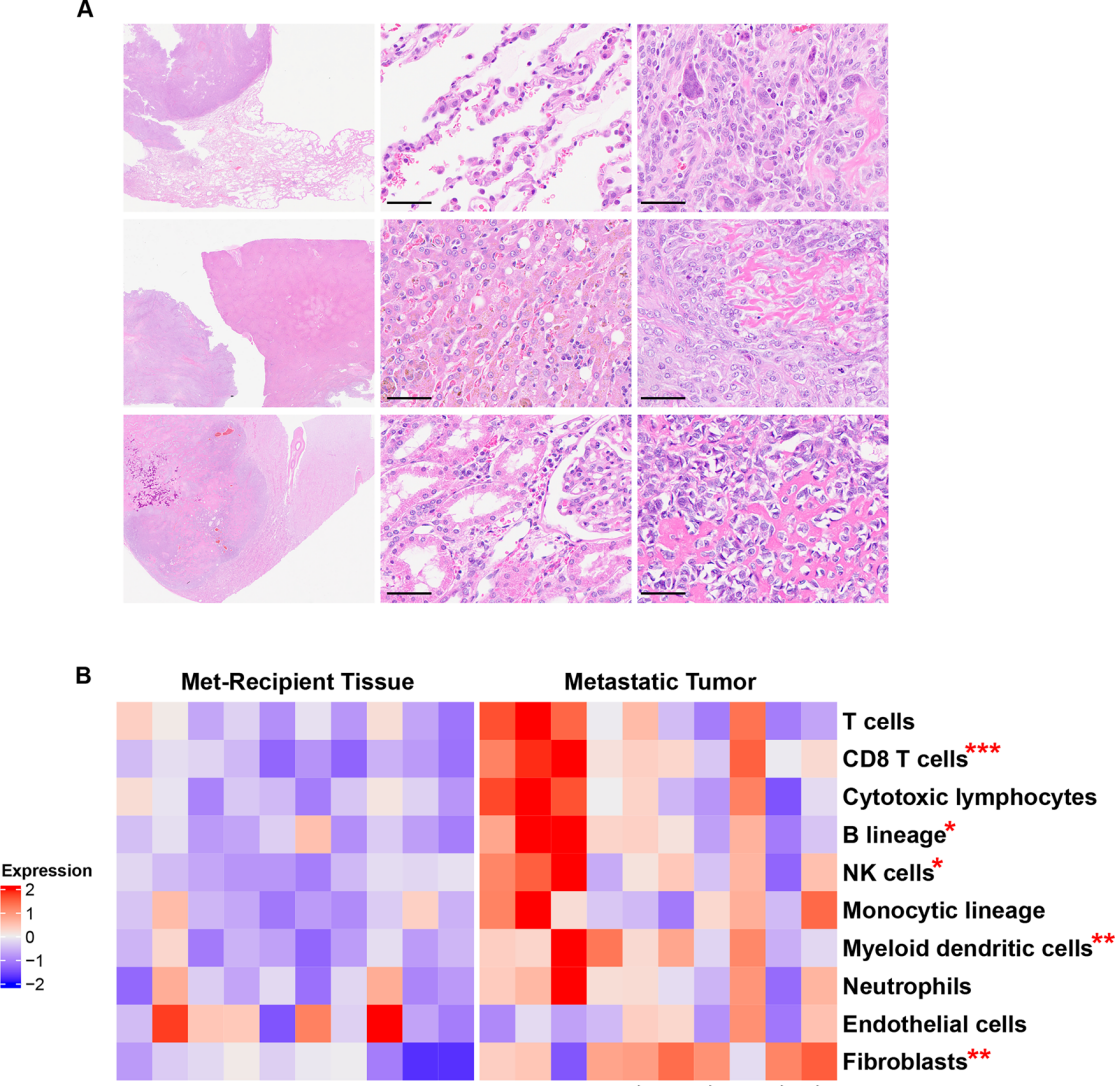
Cell Deconvolution comparison between patient-matched non-tumor and tumor lung, liver, and kidney. **A** Representative images of non-tumor and tumor tissues derived from the same canine osteosarcoma patient, (**B**) Cell deconvolution analysis demonstrating cell enrichment in patient-matched met-recipient and metastatic tumor tissues from canine osteosarcoma patients. Significant differences between cell compartments are indicated as: *p < 0.05, **p < 0.01, ***p < 0.0001. Scale bar = 50 μm.

For a subset of our met-recipient tissues, metastatic tissue data was also available (*n* = 10). We performed cell deconvolution analysis of patient-matched tumor and peritumoral tissue (Fig. 4A). This analysis identified multiple cell types that are preferentially recruited to canine osteosarcoma metastases including CD8 T cells, B cells, NK cells, and myeloid dendritic cells (Fig. 4B; Supplementary Data 2). In addition, fibroblast genes were enriched within tumor compared to the peritumoral tissue. Cell deconvolution analysis did not identify significant differences in the monocytic lineage, neutrophils or endothelial cells between peritumoral tissue and the associated metastatic lesion within this patient cohort (*n* = 10). Violin-dot plots and a table of the genes used to define each cell type are included in Supplementary Fig. 4.

### The transcriptomes of non-tumor lung, liver, and kidney in metastatic osteosarcoma overlaps with changes described in human cancer

**Fig. 5:**
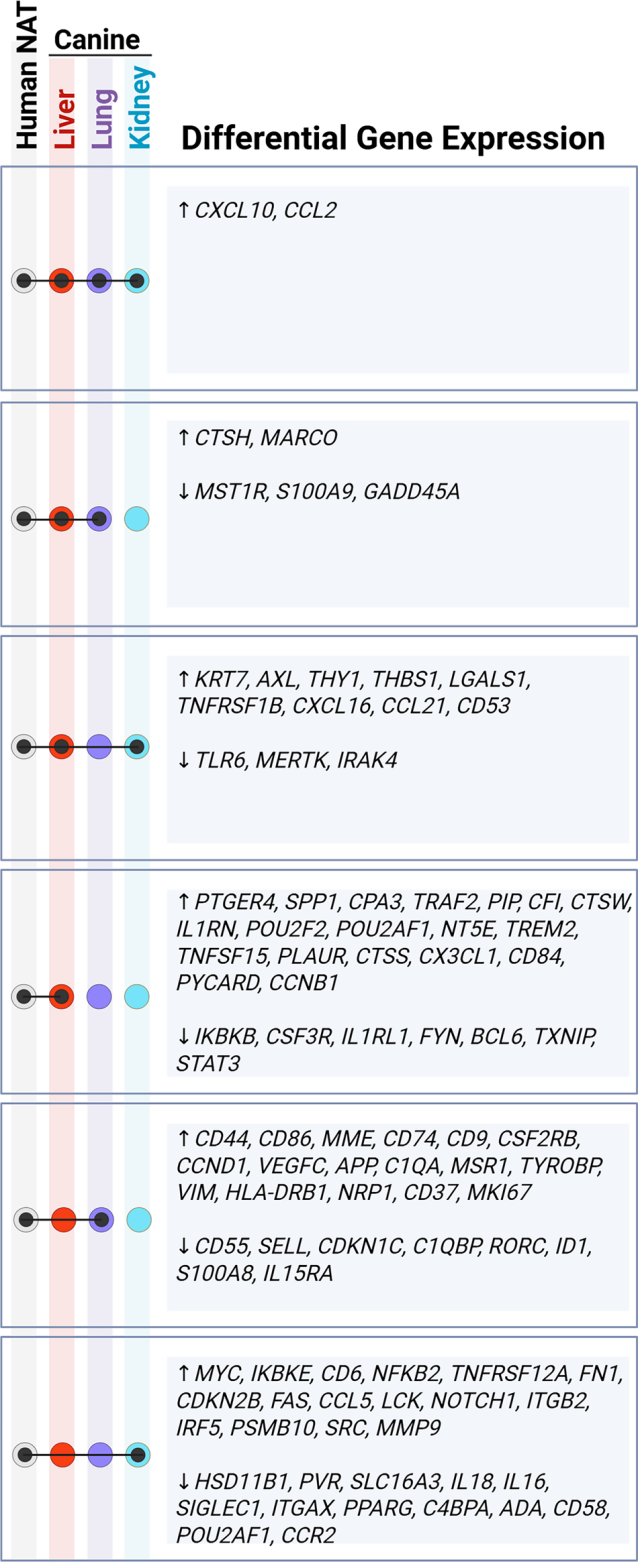
Transcriptional changes shared between canine osteosarcoma dataset and human normal tissue adjacent to tumor (NAT) study. UpSet plot listing genes shared between Human NAT study (Aran et al.), and peritumoral tissue (liver, kidney, lung) from canine osteosarcoma patients enrolled in a clinical trial. Created in BioRender.

Finally, to compare our findings to previous literature, we next compared differential gene expression between our met-recipient and met-free canine tissues to that described in a large TCGA-based study of human peritumoral tissue from multiple tumor types^13^. After filtering this list down to the genes represented in the Canine IO Panel, we compared genes up- or downregulated between met-recipient and met-free non-tumor tissues from dogs to those that were significantly differentially expressed between healthy and peritumoral tissues from human patients. When comparing to previous work in humans, we identified a larger number of upregulated compared to downregulated genes with some overlap between tissues (Fig. 5**;** Supplementary Data 2). Gene expression significantly enriched within peritumoral tissues and shared with human cancer patients include *CXCL10* and *CCL2*. No genes downregulated in met-recipient tissues across all three tissue types (lung, liver, kidney) were shared with the human dataset; however, several genes were shared across 2 of the 3 tissue types including lung and liver (*MST1R*, *S100A9*, *GADD45A*), and liver and kidney (*TLR6*, *MERTK*, *IRAK4*).

## Discussion

Post-mortem examinations are often performed as part of the clinical trial workflow for canine osteosarcoma patients. These tissue biobanks can be leveraged to pursue research questions that are difficult to address in human tissues due to a paucity of available samples, particularly in rare cancer entities such as pediatric osteosarcoma. In this study, we provide a descriptive transcriptomic analysis of metastatic TMEs in the canine model. Genes and pathways found to be enriched in met-recipient tissues were either tissue-specific or shared across tissues (lung, liver, kidney). Site-specific changes provide key insight into the pathways active in met-recipient lung, liver and kidney. For example, reduced expression of GFAP in met-recipient liver is suggestive of hepatic stellate cell activation and has been described in chronic liver disease^22^ and, although not specifically studied in osteosarcoma, may contribute to metastatic progression^27–29^. In the lung, increased expression of surfactant protein C (SP-C)^23^ and DMBT1^30^ suggests a type II pneumocyte response. These cells could contribute to a variety of functions including promotion of pulmonary fibrosis^31,32^. Examination of site-specific changes identifies key components of the metastatic osteosarcoma TME which warrant further investigation in future studies.

In contrast, shared transcriptomic changes may represent therapeutic targets with benefit across the complexity of metastatic progression in osteosarcoma patients. For example, fibroblasts are enriched across met-recipient tissues. We have previously shown that a fibroblast-rich subtype of canine osteosarcoma (IE-ECM) carries a significantly poorer prognosis both in survival and disease-free interval despite the presence of immune infiltrates suggesting that targeting ECM production might improve outcomes of specific patients undergoing immunotherapy^24^. The present study has expanded upon this finding by identifying increased expression of fibroblast genes in met-recipient, peritumoral tissues. We hypothesize that this is due to secretory factors derived from the metastatic tumor tissue and that such changes may play a role in TME remodeling that is favorable to tumor progression. As such, targeting fibrosis may provide a novel therapeutic avenue for metastatic osteosarcoma including within the lung where osteosarcoma cells have been proposed to co-opt the fibrotic wound response thereby driving pulmonary metastasis^33^.

In the present study, increased expression of CCL2 was observed in non-tumor lung, liver, and kidney of pet dogs with osteosarcoma metastases within those tissues. In addition, monocyte enrichment, as evidenced through cell deconvolution analysis, was significant in peritumoral liver but not kidney (*p* = 0.082) or lung (*p *= 0.167). Targeting the CCL2-CCR2 chemotactic axis inhibits monocyte recruitment and reduces metastatic burden in murine models^34^. Building upon this research, pharmacologic inhibition of the CCL2-CCR2 axis prevents monocyte migration and has been associated with tumor stabilization or regression in 50% of dogs in one clinical trial^35^. Critically, this canine research has led to a Phase 1 trial in human patients (ClinicalTrials.gov ID: NCT03900793); this highlights the role of tumor-bearing pet dogs in the dog-to-human pipeline for therapeutic development^36^. Our findings suggest that similar activation of this signaling axis is present across other geographically distinct sites of metastasis. Furthermore, comparison of our findings in non-tumor tissue adjacent to human cancers (Fig. 5) suggests that this signaling pathway is also observed in human cancers expanding the translational value of this therapeutic avenue across species and cancer types.

Another gene enriched across peritumoral tissues of metastatic osteosarcoma is CXCL10; CXCL10 mediates signaling between tumor and stroma and contributes to multiple processes including leukocyte trafficking, angiogenesis, and metastatic progression^37,38^. This gene enrichment is also shared within non-tumor tissue adjacent to multiple cancer types in human patients^13^. Furthermore, high circulating CXCL10 has been associated with poor prognosis in pediatric osteosarcoma patients independent of metastasis at diagnosis^39^. Cells which we found to be enriched within the peritumoral tissue of lung, liver, and kidney metastases in this study are also frequent sources of CXCL10 including monocytes and fibroblasts^40–42^.

Although we identified approximately 100 genes with similar alterations as described in human tissues^13^ (Fig. 5), over 200 genes identified in human tissues were not similarly dysregulated between met-free and met-recipient lung, liver, or kidney in canine osteosarcoma patients (Supplementary Data 2). There are several key differences between these studies that should be considered. First, our peritumoral tissues were obtained in the context of osteosarcoma metastases while the previous study evaluated primary tumors from multiple cancer types from TCGA. Second, our met-recipient and met-free tissues were all derived from osteosarcoma-bearing pet dogs. Therefore, differences in gene expression may be due to cancer type (e.g., osteosarcoma vs. pulmonary adenocarcinoma or hepatocellular carcinoma), process (e.g., primary cancer vs. metastasis), or species and source (canine clinical trial vs TCGA/GTEx). Shared transcriptional alterations highlight the valuable role played by osteosarcoma-bearing pet dogs in the development of TME-targeting therapeutics. In addition, we provide added strength that shared changes are unlikely to be solely due to field cancerization as the study samples were collected from metastatic sites. Finally, while previous work suggests that alterations in the peritumoral transcriptome may be essential for tumorigenesis^13^, the present study expands upon these findings to suggest that these genes may also play a role in metastatic disease.

One weakness of our study is the lack of data pertaining to where tissues were geographically collected in relation to the tumor. For the kidney, all samples were collected from annotated non-tumor tissue adjacent to tumor because records did not indicate whether met-free sections of kidney were from a kidney that contained a metastatic lesion or the contralateral kidney which may have been entirely met-free. Lung samples were taken from grossly normal regions. The liver dataset included both sample types. The proximity of non-tumor kidney samples may be one explanation for the higher number of significantly different genes in the kidney over lung. One way that we aimed to address this was through the evaluation of patient-matched samples (Supplemental Fig. 1) which were taken either adjacent or distant to the tumor sample thereby identifying genes that may be more affected by distance to the tumor lesion. Another factor which may have impacted our ability to identify shared alterations across tissue locations is preservation technique (Supplementary Data 1). In contrast to kidney and liver, the lung cohort was derived from frozen samples because RNA of sufficient quality and quantity could not be obtained from FFPE lung. This difference should be considered when evaluating these results and comparing alterations across tissue sites; however, the propensity of osteosarcoma cells to metastasize to the lungs in both canine^9^ and human patients^10^ underscores the importance of including this dataset in this study.

Another limitation of this study is the variability in gene expression between individuals within a group. This variability is important to consider when aiming to identify gene signatures with predictive value. To this point, this work serves primarily as a descriptive transcriptomic analysis of the metastatic TME of osteosarcoma. Future work with larger cohorts of outcome-linked patients or functional studies are required to provide clinical translation for the findings described herein. In addition, although we specifically aimed to interrogate changes associated with the presence of a metastatic lesion, additional studies comparing osteosarcoma patients to non-tumor bearing controls would offer additional insight into the systemic impact of osteosarcoma and chemotherapy on non-tumor tissues. Finally, although the NanoString IO panel is a valuable resource for querying degraded RNA, future spatial transcriptomics or single cell RNA sequencing studies are well positioned to further interrogate stromal biology in the peritumoral tissue.

In conclusion, we have identified both shared and tissue-specific changes in met-recipient non-tumor lung, liver, and kidney that should be considered when investigating the TME’s role in metastatic progression and therapeutic response. In addition, we have documented similarities to that which has been described in humans with other tumor types. These findings expand our understanding of the transcriptional and cellular landscape of the peritumoral TME of metastatic osteosarcoma and improve our ability to develop therapeutic strategies that overcome boundaries within it for the benefit of osteosarcoma patients.

## Methods

### Tissue samples

Formalin-fixed liver, formalin-fixed kidney and frozen lung samples were collected during necropsy of pet dogs with a confirmed diagnosis of appendicular osteosarcoma that participated in a multisite prospective clinical trial^21,43,44^. Diagnosis of osteosarcoma metastases was based on gross and histologic examinations performed by veterinary anatomic pathologists at Comparative Oncology Trials Consortium (COTC) institutions. All canine clinical trial activities were carried out in accordance with relevant guidelines and regulations, with study reporting according to ARRIVE guidelines. Each participating COTC institution’s Animal Care and Use Committee (ACUC) approved the clinical protocol. Written informed consent from all dog owners was obtained prior to enrollment in the COTC021/022 trials^21^. Non-tumor tissues derived from osteosarcoma patients with and without metastases are referred to as met-recipient and met-free tissues, respectively. For liver and kidney, met-recipient tissues were collected from formalin-fixed paraffin-embedded (FFPE) tissues that either did not contain tumor tissue or were annotated to exclude tumor tissue (Fig. 1). With the exception of a comparison between patient-matched samples (Supplementary Figs. 1–2; Fig. 4), all kidney samples were enriched for cortical tissue. A single pair of patient-matched lung samples distant and adjacent to a pulmonary metastasis was collected from FFPEs; the remaining lung samples were frozen non-tumor tissue collected at the time of necropsy. Additional information about the samples can be found in Supplementary Data 1 including sample location, preservation method, and DV200.

### NanoString IO panels

RNA isolation from frozen lung was completed by NCI’s CCR Genomics Technology Laboratory. RNA isolation from FFPE tissue was completed by the Molecular Histopathology Laboratory (NCI) as described previously^24^. Non-tumor tissue proximal to osteosarcoma metastases was obtained via hand macro-dissection. All other samples were collected via scrolls. RNA quality was assessed by Agilent Tape station to identify samples for which at least 50% of the RNA fragments were greater than 200 nucleotides (DV200 ≥ 50%). All samples were loaded onto NanoString Canine Immuno-oncology panels (100 ng total for frozen lung; 150 ng for liver and kidney). All hybridizations were 17 to 22 h long, and all counts were gathered by scanning on HIGH mode for 280 fields of view per sample. RNA was profiled using the Canine IO Panel and analyzed with the nCounter System (NanoString Technologies; RRID:SCR_023912), according to the manufacturer’s protocol.

### Preparation and preprocessing of the tissue dataset

To mitigate technical variability across the tissue samples, a two-step normalization procedure was conducted using the nanostringr (v.0.4.2) platform^45^. The positive control normalization approach was employed to account for all platform-associated sources of variation. However, this type of normalization does not address differences in sample input between technical or biological replicates. Consequently, sample-specific correction within each sample lane was performed using CodeSet content (housekeeping gene) normalization. 835 of 869 genes were used for the downstream analysis.

### Differential expression, gene set enrichment, and cell deconvolution analyses

The normalized raw count data and the sample grouping information for each tissue site were provided as input to the edgeR (v.4.2.1) software^46^. The “Upper quartile” normalization method was applied to calculate normalized pseudo-counts for differential expression analysis across the sample groups. For each group, all genes were ranked based on their estimated log fold change in expression, as determined by edgeR. Genes with a high positive log fold change (i.e., up-regulated) were ranked at the top, while those with a high negative log fold change (i.e., down-regulated) were ranked at the bottom. This ranked list of genes for each group was then used as input to the standard Gene Set Enrichment Analysis (GSEA) pipeline, implemented in the clusterProfiler (v4.12.1) package^47,48^ using default parameters, to assess the relative enrichment of hallmark pathways in each group. The quantification of differentially expressed genes was performed to identify significant changes in gene expression across the tissue samples. Comparative deconvolution analysis was performed across multiple tissue samples using the MCP counter (v1.2.0) tool with default parameters^49^. The normalized gene expression data were input into MCP counter to quantify the relative abundances of specific cell types, including CD8 + T cells, CD4 + T cells, cytotoxic lymphocytes, B cells, NK cells, and cells from monocytic and myeloid lineages which are represented using ComplexHeatmap (v.2.20.0).

### Signature scores for shared TME Genes

To further investigate gene expression within the osteosarcoma TME, we examined publicly available, preprocessed spatial transcriptomics from human osteosarcoma metastases with sufficient peritumoral tissue (lung; *n* = 6)^25^ and single-cell RNA sequencing from canine primary osteosarcomas (bone; *n* = 6)^26^. Cell type deconvolution was performed for human samples using a method described previously^25^, employing a comparable set of cell-type specific marker genes. Signature scores were calculated for the shared genes of interest (CCL2, COL1A1, COL3A1, and CXCL10) for each cell-type, and incorporated into the Seurat object metadata via the AddModuleScore_UCell function^50^. A generalized linear model (GLM) was fitted for each gene signature score across annotated cell types.

## Supplementary information

Supplementary Information

Supplementary data 1

Supplementary data 2

Summary

## Data Availability

The canine clinical trial datasets generated and/or analyzed during the current study are available in the GEO database (RRID:SCR_005012; accession ID: GSE309676). Source data is available in Supplementary Data 2.

## Code availability

Codes are deposited at GitHub (https://github.com/Anjaligarg006/NAT.git).
