# Supplementary material for: Comprehensive analysis of non-tumor lung, liver, and kidney transcriptomes in canine metastatic osteosarcoma
Source: Commun Biol. 2026 Mar 23;9:741. doi: 10.1038/s42003-026-09870-x (PMC13230841; doi:10.1038/s42003-026-09870-x)
Supplement: Supplementary file 1 — Supplementary Information [file 42003_2026_9870_MOESM1_ESM.pdf]

Supplemental Figure 1

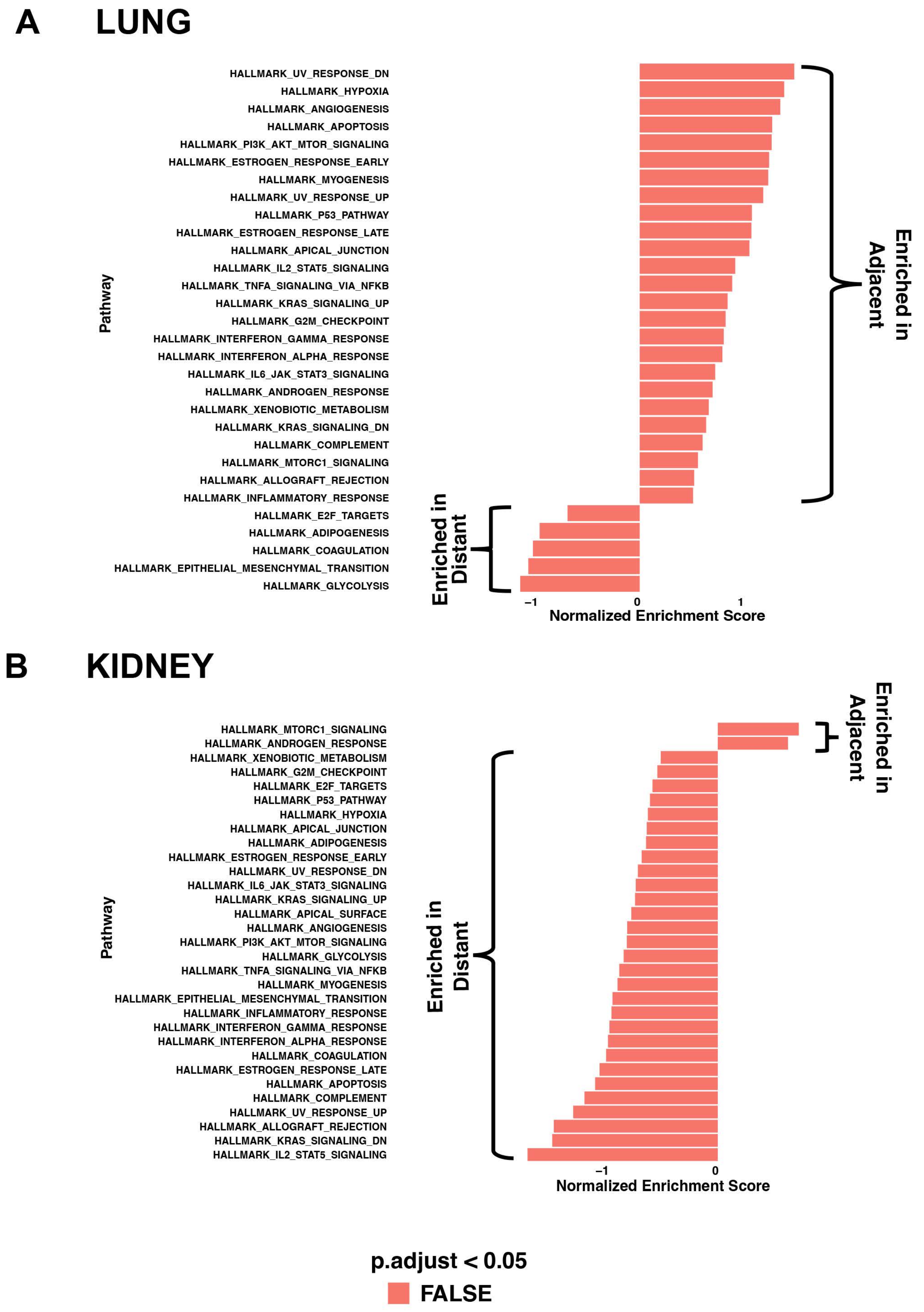

Supplemental Figure 1. Pathway enrichment in regions either adjacent to or distant from metastatic lesions within the (A) Lung and (B) Kidney

Supplemental Figure 2

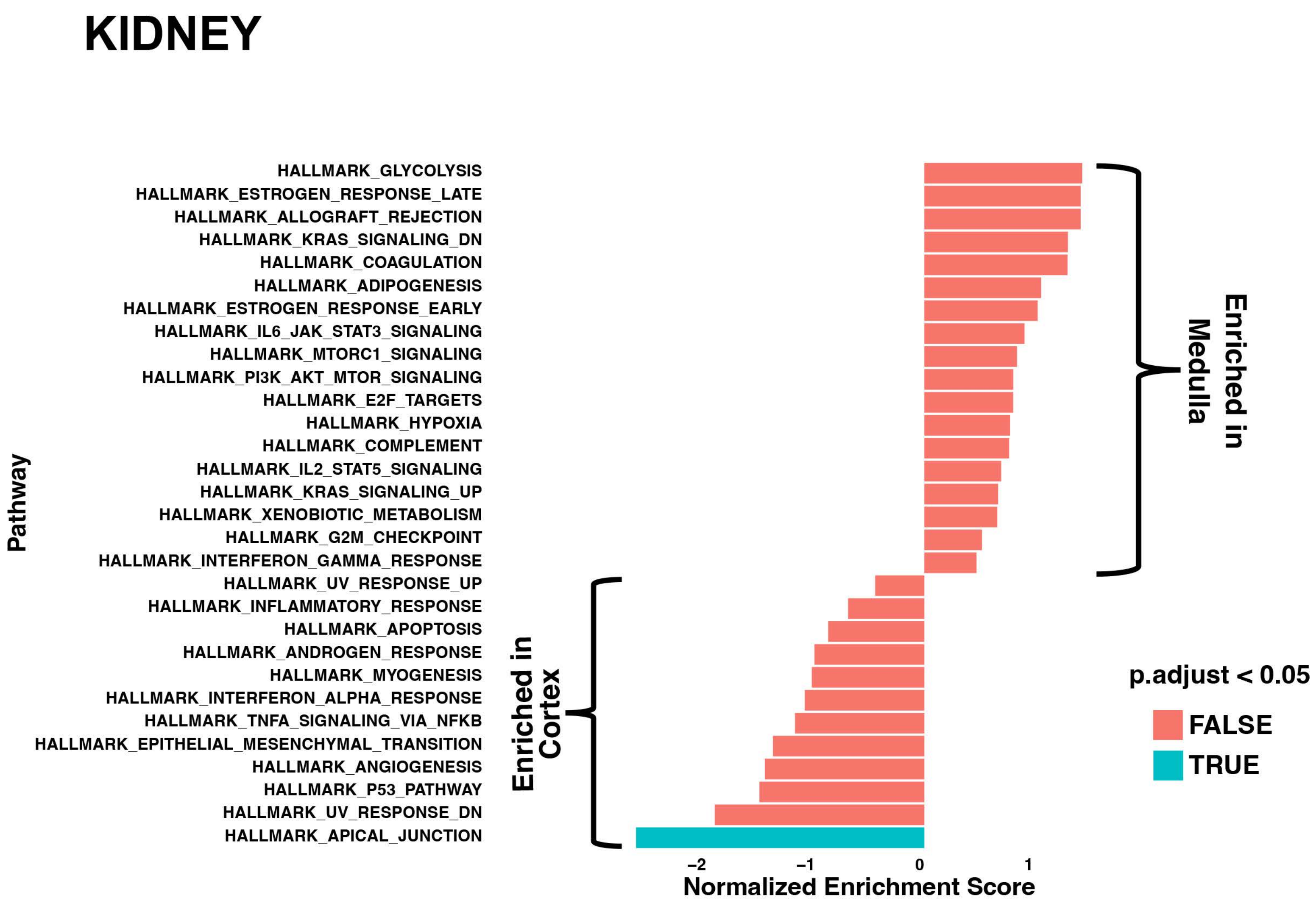

Supplemental Figure 2. Comparison of pathway enrichment between renal cortex and renal medulla

Supplemental Figure 3

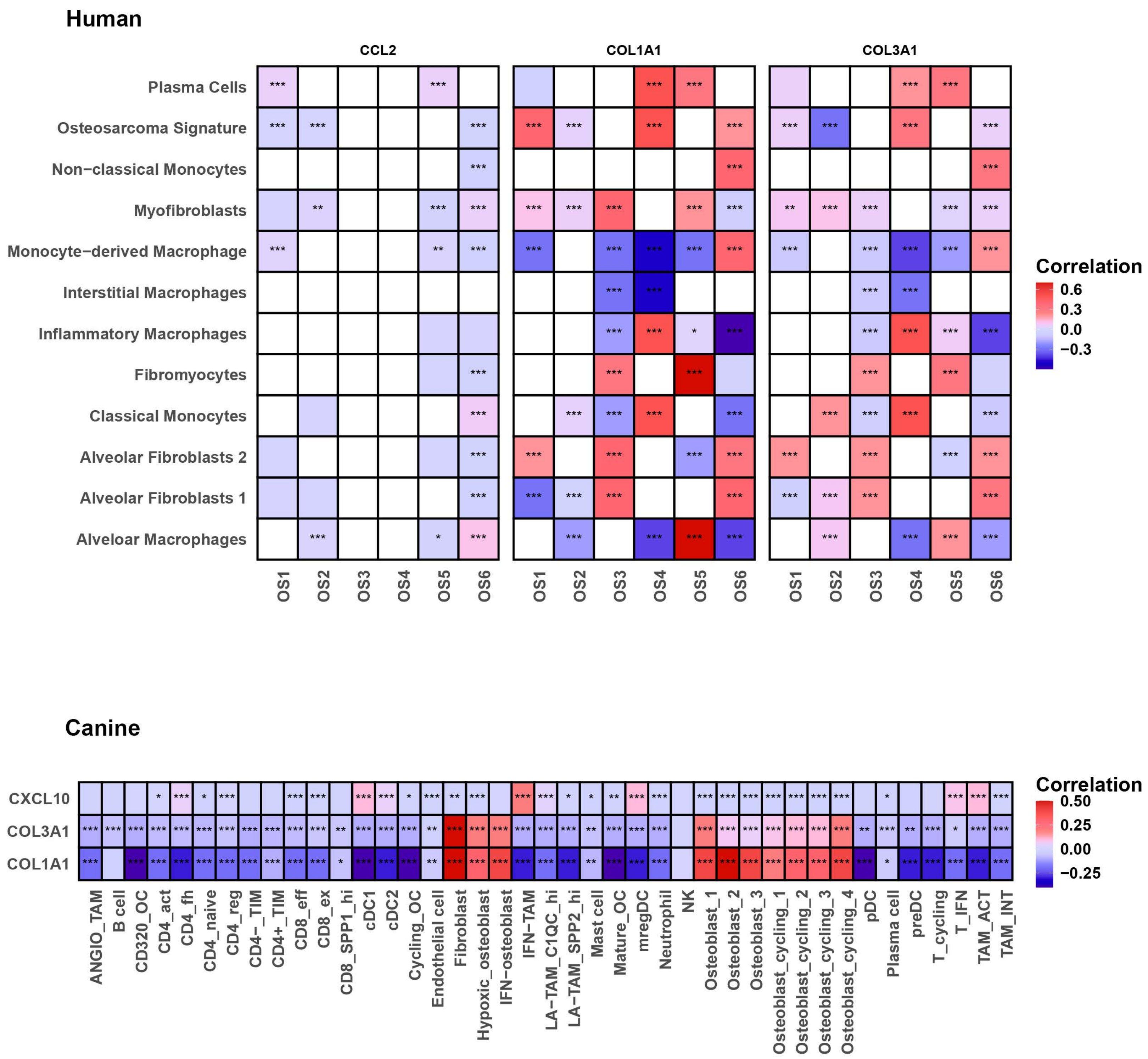

Supplemental Figure 3. Correlation between CCL2, COL1A1, COL3A1, and CXCL10 and cell types derived from publicly available human and canine osteosarcoma datasets.

Supplemental Figure 4

Cell Type	MCP Counter Gene List
T cells	*CD28, CD3D, CD3G, CD5, CD6, CHRM3-AS2, CTLA4, FLT3LG, ICOS, MAL, MGC40069, PBX4, SIRPG, THEMIS, TNFRSF25, TRAT1*
CD8 T cells	*CD8B*
Cytotoxic lymphocytes	*CD8A, EOMES, FGFBP2, GNLY, KLRC3, KLRC4, KLRD1*
B lineage	*BANK1, CD19, CD22, CD79A, CR2, FCRL2, IGKC, MS4A1, PAX5*
NK cells	*CD160, KIR2DL1, KIR2DL3, KIR2DL4, KIR3DL1, KIR3DS1, NCR1, PTGDR, SH2D1B*
Monocytic Lineage	*ADAP2, CSF1R, FPR3, KYNU, PLA2G7, RASSF4, TFEC*
Myeloid Dendritic Cells	*CD1A, CD1B, CD1E, CLEC10A, CLIC2, WFDC21P*
Neutrophils	*CA4, CEACAM3, CXCR1, CXCR2, CYP4F3, FCGR3B, HAL, KCNJ15, MEGF9, SLC25A37, STEAP4, TECPR2, TLE3, TNFRSF10C, VNN3*
Endothelial Cells	*ACVRL1, APLN, BCL6B, BMP6, BMX, CDH5, CLEC14A, DIPK2B, EDN1, ADGRL4, EMCN, ESAM, ESM1, FAM124B, HECW2, HHIP, KDR, MMRN1, MMRN2, MYCT1, PALMD, PEAR1, PGF, PLXNA2, PTPRB, ROBO4, C, SHANK3, SHE, TEK, TIE1, VEPH1, VWF*
Fibroblasts	*COL1A1, COL3A1, COL6A1, COL6A2, DCN, GREM1, PAMR1, TAGLN*

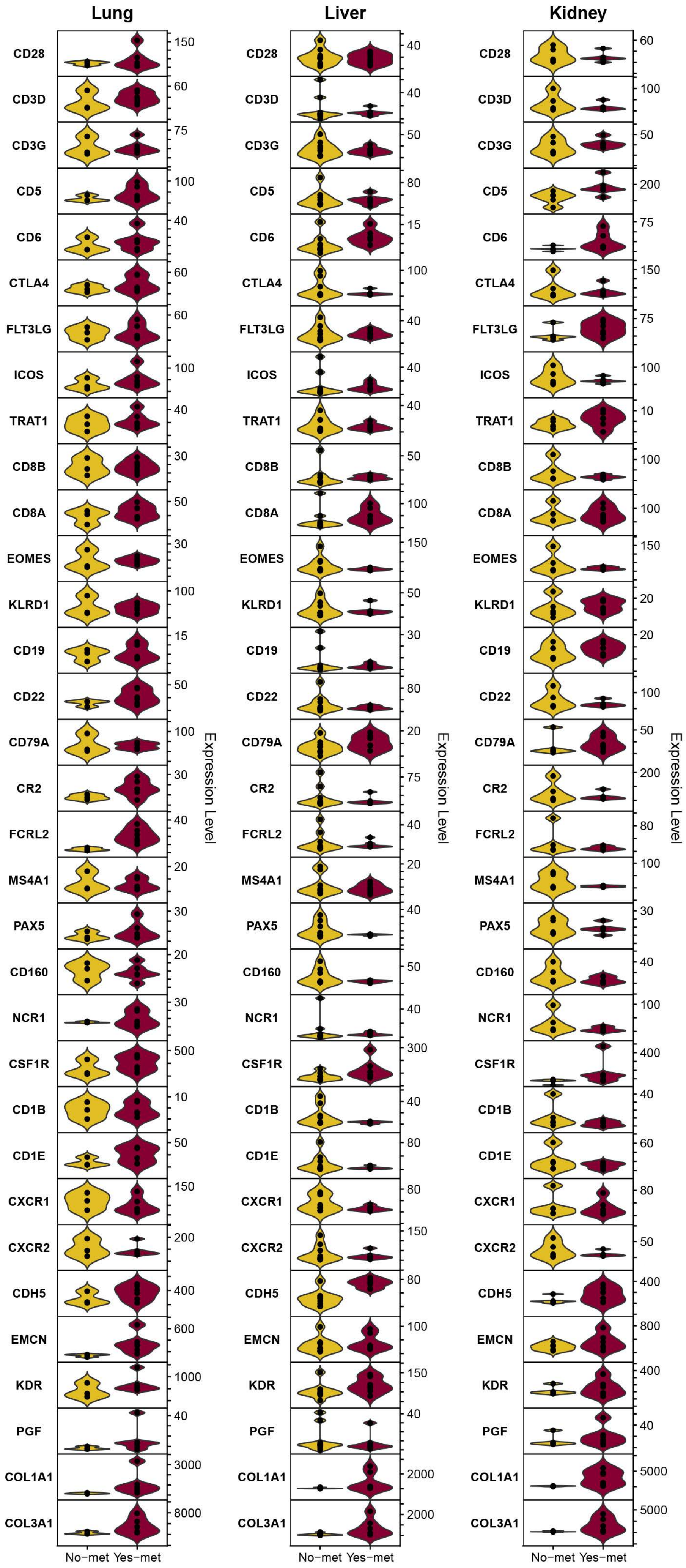

Supplemental Figure 4. MCP counter gene list and violin-dot plots for genes used to identify specific cell types within canine lung, liver, and kidney.
